# Monitoring the effects of land cover change on the supply of ecosystem services in an urban region: A study of Santiago-Valparaíso, Chile

**DOI:** 10.1371/journal.pone.0188117

**Published:** 2017-11-14

**Authors:** Claudia Montoya-Tangarife, Francisco de la Barrera, Alejandro Salazar, Luis Inostroza

**Affiliations:** 1 Instituto de Geografía, Pontificia Universidad Católica de Chile, Santiago, Chile; 2 Centro del Desarrollo Urbano Sustentable, Pontificia Universidad Católica de Chile, Santiago, Chile; 3 Facultad de Arquitectura, Urbanismo y Geografía, Universidad de Concepción, Concepción, Chile; 4 Institute of Geography, Ruhr-University Bochum, Bochum, Germany; 5 Universidad Autónoma de Chile, Santiago, Chile; University of Missouri Columbia, UNITED STATES

## Abstract

Mankind’s quest for well-being results in continuous pressure to transform landscapes, with said transformation driven by land use changes, urbanization, production activity, and protective measures in addition to climate variability and other environmental drivers. The relationship between anthropogenic landscape changes and the provision of ecosystem services (ES) is a topic of increasing interest in Latin America. In Chile, land cover changes due to increased urbanization and forestry, and expansion of agricultural land, in addition to conservation initiatives as a part of land planning, have been intensive in the last few decades. In this study, the effects of anthropogenic landscape changes on the supply of ES were analyzed for the urban region of Santiago-Valparaiso (Chile) using a method based on expert consultation and land cover change assessment. A pool of experts scored the potential of specific land covers to provide certain ES. The results enabled calculation and mapping of changes in the potential of the landscape to supply ES. The aforementioned changes over a period of 15 years were evaluated. The results indicate a tenuous balance between positive and negative changes to the supply of ES derived from land cover changes. Understanding and reporting how these processes occur in urban regions contributes to the conservation of valuable landscapes through spatial planning tools, especially in areas close to housing developments and sensitive ecosystems.

## 1. Introduction

Ecosystem services (ES), defined as “the benefits that people obtain from ecosystems” ([1]: p.630, [2]: p.40), are fundamental for society [3, 4]. ES improve human well-being and environmental quality in several ways, for example, by capturing air pollutants and/or providing fresh water (regulating ES), as well as through economic benefits such as those obtained from access to goods for subsistence or for the generation of wealth (provisioning ES). Additionally, ES also offer cultural services such as aesthetics, contact with nature, or recreation opportunities (cultural ES) [3, 4, 5]. At the same time, several anthropogenic activities and drivers, such as forest plantations, mining, agriculture, and urbanization, directly and indirectly transform landscapes [6–11]. Even the protected status of landscapes makes them more attractive to visitors and dwellers, there by triggering landscape changes [12]. Indeed, changes in ecosystem dynamics in populated areas are mostly driven by anthropogenic factors [13]. Such landscape changes are not only caused by a small set of decision makers and drivers but also by wildfires, dispersed disturbances, socio-natural events, climate variability, land management, etc. [14, 15]. Globally, two of the most extensive drivers of landscape changes are: 1) the demand for primary and secondary housing [9, 16, 17] and 2) the expansion of the forestry and agricultural industries [18, 19, 20]. Landscape change, in turn, affects the supply of ES [21, 22, 23].

Landscape changes are usually analyzed via land use/land cover information obtained from remote sensing or ad-hoc spatial data sets. Land cover serves as a proxy indicator of the provision of ES, thereby enabling the analysis of trade-offs between land cover changes and the supply of ES (e.g. [2, 3, 24–31]). Whereas research on ES has greatly increased over the last few decades (e.g. [3, 24, 32, 33]), its direct use in policy-making and spatial planning remains challenging. For instance, limitations in both the availability of ES studies and integration of strategies with decision-making processes present some of the associated challenges [5, 34, 35]. Research on land cover and ES requires good quality spatial information, which is scarce for Latin American countries [22, 27, 29, 36]. To date, despite their great potential in supporting spatial planning, studies regarding land cover changes and their effects on the provision of multiple ES in Chile are still scarce [37]. Moreover, research concerning land cover changes has focused on promoting forestry and the conservation of native forests without considering the ES those areas provide [37]. Recent research indicates high rates of loss of native forest and expansion of forestry in central south Chile [38, 39, 40], a situation that poses a threat to fundamental ES. Research on ES has been primarily focused on the economic (monetary) valuation of ES, which has been used for comparisons among alternatives for project development or for assessing water production or pollution [33, 37, 41, 42].

Urban regions are highly dynamic landscapes that encompass extensive metropolitan areas, satellite cities, and dispersed housing and are usually bisected by a river, crops, and/or native ecosystems [43]. In Latin America, especially in Chile, urban regions present a great challenge for spatial planning owing to three main reasons: 1) the pace and spatial pattern of urban expansion of metropolitan areas [44]; 2) the absence of ecological dimension, highly relevant at landscape scale, in standard urban planning; and 3) the sheer number of actors involved in the planning process. An ES framework could be of great help in dealing with these challenges [45].

This paper presents research in which an adapted method from Burkhard [25] was applied to assess the effects of land cover changes on the supply of ES in the urban region of Santiago-Valparaiso (Chile). GIS spatial analyses and expert consultation techniques were used to ascertain spatiotemporal changes in the supply of ES, and in particular, to inform spatial planning. The specific objectives, therefore, were: 1) To develop an adaptation of the land cover and expert consultation based methods for assessing the potential supply of ES, 2) to ascertain the variations in ES over time, identifying the land cover changes with higher effects on the supply of ES and 3) to analyze the changes in the spatial distribution of ES in the Santiago-Valparaiso urban region.

## 2. Materials and methods

### 2.1 Santiago-Valparaíso urban region

**Fig 1:**
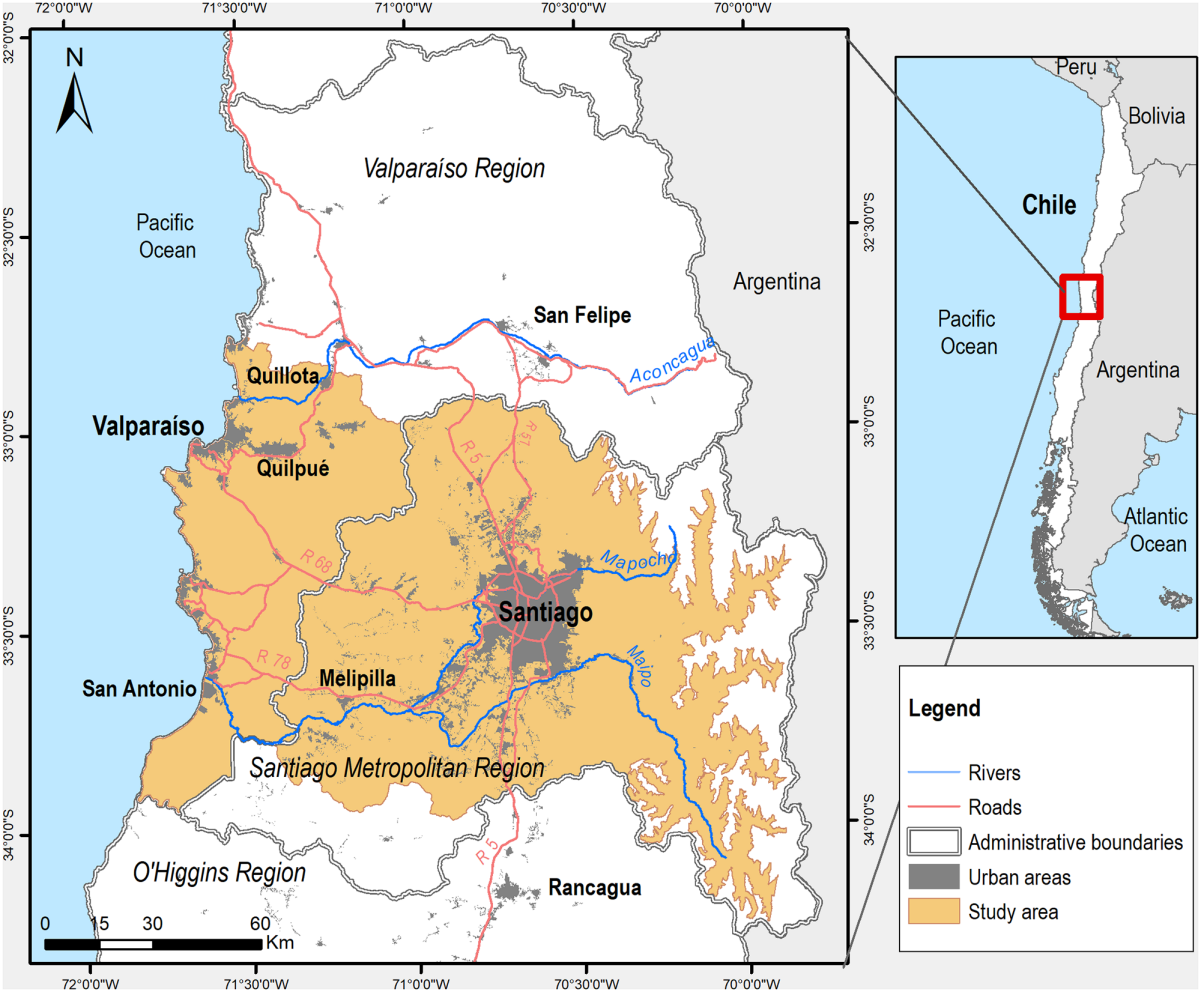
Study area: Urban region of Santiago and Valparaíso, Chile (67 municipalities).

In this study, a dynamic and highly populated Latin American urban region was selected. The study area covers 15,655 km^2^ (Fig 1), comprising the administrative boundaries of 67 municipalities, and integrates the functional and spatial interaction of two regions located in central Chile, Santiago and Valparaiso. These two regions have undergone rapid urbanization and population growth [17, 46], reaching 7,881,273 inhabitants, which is equivalent to 50.3% of the national population [47]. Projections for 2015, envisaged a 7% increase, resulting in 9.1 million inhabitants [47]. Inaccessible areas (over 3,300 m.a.s.l.) in the east, mainly consisting of steppes and bare soils, were left out of the analyses. The urban region is a landscape characterized by two orographic systems parallel to the ocean coastline, i.e., the Andes and the coastal range. The landscape is crossed by rivers that form a coastal system of beaches, dunes, and wetlands. Other ecosystems include natural vegetation types adapted to droughts, humid conditions in ravines, and geographical elevation [48, 49]. Owing to a regime of frequent perturbations, several degraded ecosystems, such as open thorny shrublands and grasslands with scarce vegetation, are also found. The mosaic is completed with agricultural fields composed mostly of vineyards and fruit trees, forest plantations of exotic species, and different types of urban areas.

### 2.2 Land cover classification

**Fig 2:**
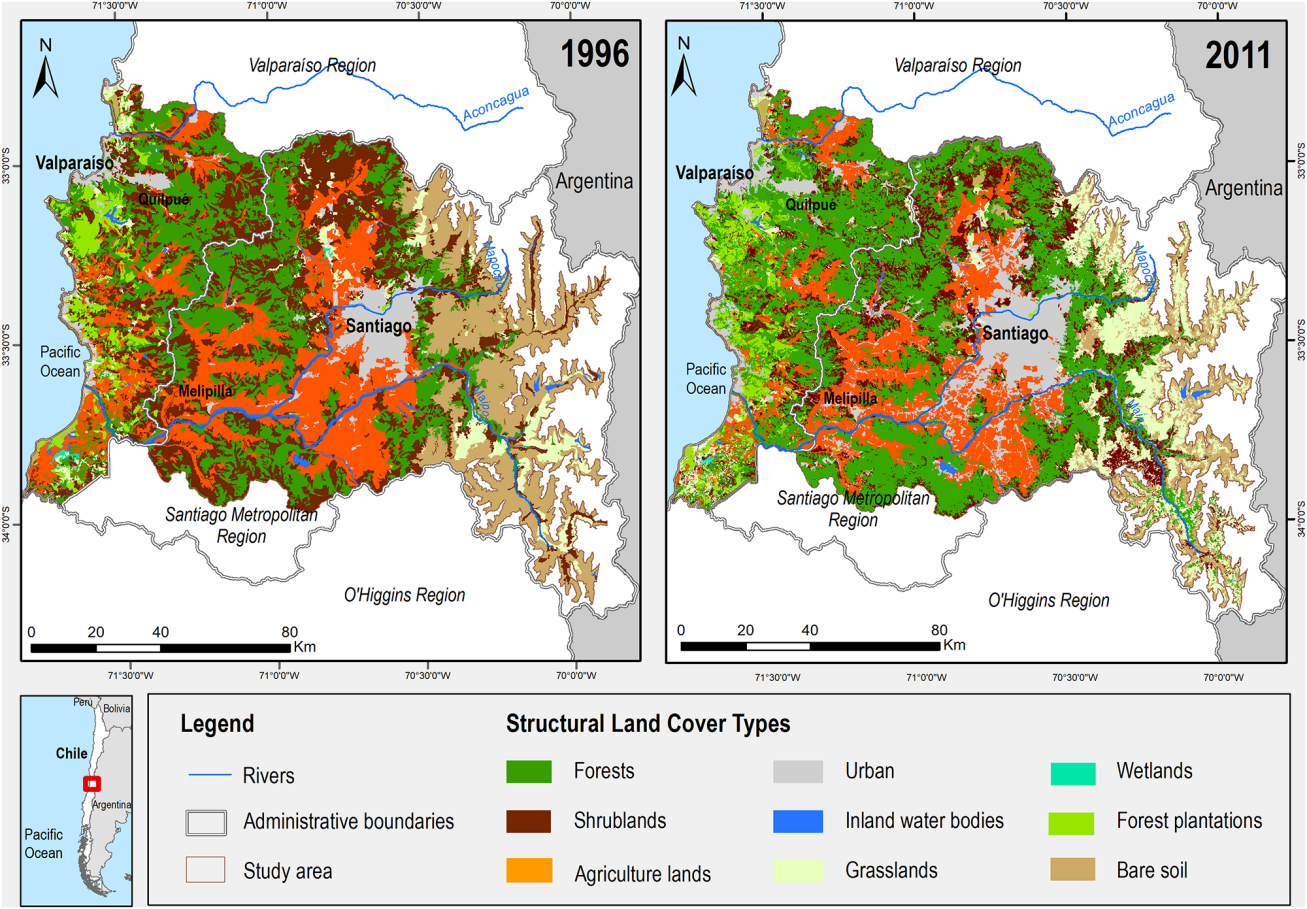
Land cover of the urban region in 1996 and 2011 post analysis of the official datasets [50, 51].

Landscape changes were assessed using a short list of simplified land cover types to focus the analysis on structural changes. The spatial data set was derived from the official land cover database (also known as regional cadasters of native forest and land uses), which originally provided official data from 1996 and was later updated in 2011 [50, 51]. This database has been used in several studies carried out for different regions of Chile that mainly involved analyzing trends in land cover changes (e.g. [39, 40, 52]). Official databases often have limitations, such as the use of different criteria each year to classify land covers [38]. To cope with such limitations and reduce inconsistencies and uncertainties in temporal changes, land covers were merged into structural classes. The most critical discrepancy was observed for forest type, which was classified as a forest by the method used in the official land cover database category for 1996 but as a dense shrubland in the previous official dataset [50]. To overcome this problem, both classes were combined into a single category renamed “forests and shrublands”. Considering the ecological structure of the ecosystem in the study area, this was a consistent methodological decision. The official database contained 66 detailed land cover types, including only one urban type and many types for forest and scrub coverage. These 66 detailed land cover types were grouped into nine simplified structural land covers that account for the scale of the analysis and the land cover types as described by Schultz [46] for central Chile (Table 1). The official dataset classification is presented in Fig 2. Table A in S1 Appendix presents the integration of the detailed land cover classes into the structural land covers.

**Table 1:** Land cover types grouped into structural land covers.

Structural land cover	Groups of land cover types
Forests	Mature forests (native species and mixed)* Dense sclerophyllous shrublands Dense deciduous shrublands
Shrublands	Sclerophyllous shrubs (medium to low densities) Succulent species (large patches) Xerophyte shrubs
Agricultural lands	Permanent agricultural activities Vineyards Fruit farms
Forest plantations**	Pine monocultures Eucalyptus monocultures
Urban	Residential Commercial Industrial Airports Others***
Inland water bodies	Rivers Lakes Ponds
Bare soils	Degraded soils Freshly harvested croplands Beach rocks Dunes
Grasslands	Steppes Annual grasslands Meadows Riverbank herbaceous vegetation
Wetlands	All naturally flooded lands

*All types of forest.

**Allocated to forest production.

***Large sites with artificially sealed surface.

From an ecosystem perspective, these nine proposed structural land covers, namely forests, shrublands, agricultural lands, urban areas, inland water bodies, grasslands, wetlands, forest plantations, and bare soils (Table 1), have very different vertical and horizontal structures, thereby implying different capacities for the supply of ES. This land cover simplification is ad-hoc for the landscapes of central Chile, which are characterized by extensive shrublands. In some cases, these shrublands can become forests through the process of ecological succession or stay as dense shrublands, the latter being considered the more mature ecological status. Both forests and dense shrublands are highly dense in biomass and have rich and deep soils. By contrast, degraded shrublands or primary shrublands (at an early stage of ecological succession) are less densely vegetated and have poor soils. This simplification made it possible to create a consistent landscape categorization that takes into account the scale of the analysis and facilitates expert consultation.

### 2.3 Ecosystem services

A list of 19 ES existing in the region was compiled by adapting the Common International Classification of Ecosystem Services (CICES) [29] and the work of Burkhard [53]. From this initial list, a matrix was developed to assess the potential supply of ES for each of the nine structural land covers. The potential supply of ES can be defined as the “hypothetical maximum performance of a given land cover to supply a specific ES” ([54]: p.18). Of the 19 selected ES, nine were assigned to the regulating services group, six to the provisioning services group, and four to cultural services. Table 2 presents the ES used in this research and compares them to those employed in other similar studies [22, 53, 55, 56]. In addition, a combined matrix was built to normalize the values provided in each of these studies and to correlate the results found in the current study with those in the studies mentioned above.

**Table 2:** Ecosystem services used in this study and those used in the reviewed literature [22, 5,3, 5,5, 5,6].

Ecosystem services groups	This research	Burkhard et al., 2014	Kopperoinen et al., 2014	Koschke et al., 2012	Vihervaara et al., 2010
**Regulating services**	Global climate regulation	Global climate regulation	Local and regional climate regulation	Climate regulation (global)	Local and regional climate
	Local climate regulation	Local climate regulation	-	Climate regulation (local)	-
	Hydrological regulation	Water flow regulation	Water flow regulation	Water (balance) regulation	Flood prevention
	Control of pests and diseases	Pest and disease control	-	-	-
	Regulation of air quality	Air quality regulation	Air flow regulation	Clean air provision	-
	Regulation of soil erosion	Erosion regulation	-	Soil erosion protection	Erosion prevention
	Regulation of nutrient and soil formation	Nutrient regulation	-	-	Nutrient cycling, soil formation
	Surface water purification	Water purification	Water quality regulation	-	-
	Pollination and seed dispersal	Pollination	Pollination		Pollination
**Provisioning services**	Crops	Crops	-	-	-
	Livestock and poultry production	Livestock (domestic)	Produced terrestrial plants and animals for food	-	-
	Fisheries and aquaculture	Fish, seafood and edible algae, Aquaculture	Aquaculture products	-	Fish
	Wood, fuelwood, and fibers	Fiber, timber, wood fuel	-	Food and fiber, wood/timber	Wood
	Fresh water	Freshwater	Water for human consumption and agricultural use	Clean water provision	Water
	Medicinal plants	Biochemicals and medicine	Biotic materials	-	Medicines
**Cultural services**	Recreation, tourism, and sport in natural settings	Recreation and tourism	Landscape character for recreational opportunities	Recreation and ecotourism	Recreation and silence
	Aesthetics	Landscape aesthetics and inspiration	Aesthetics and heritage	Aesthetic	Esthetic landscape
	Existence	Religious and spiritual experience	-	-	-
	Research and education	Knowledge systems	Scientific and educational information and knowledge	-	-

### 2.4 Expert consultation

Expert consultation has been used as a frequent alternative to estimate the provision of ES, especially in contexts lacking good quality data (e.g. [22, 42, 53, 55, 57–63] among others). This methodology is fast, practical, and efficient compared to procedures of greater complexity that require intensive work as well as large data sets to calculate ES supply. The expert consultation assessment was based on a matrix consisting of ES in columns and land covers (as equivalents to ecosystem elements; Table 1) in rows. Experts were contacted and asked to score the potential of each ecosystem to provide specific ES using a qualitative scale from 0 to 5, where zero means no potential and five means maximum potential.

International researchers from different disciplines with expertise in the central Chilean landscape and its dynamics, as well as experience in ES evaluation, were contacted. Only a select group of experts in Chile fulfills these requirements. From the 22 experts consulted originally, 15 answered the survey. This final group of experts represents different disciplines: geography (6), agronomy (1), ecology (5), forest engineering (2), and sociology (1). All experts hold a PhD degree, have publications in the field, and are involved in planning or policy-making. Each expert was given detailed instructions on how to complete the matrix with qualitative scores.

An average for all responses was calculated to get a single assessment matrix that was later used to calculate the respective supply of ES. To calculate the total provision of ES and to test the sensitivity of the grouping, two options were explored: 1) equally weighting all ES and 2) assigning different weights to each group.

Additionally, the standard deviation of the answers was calculated to ascertain variability. Assuming that the smaller the standard deviation, the more the experts are in agreement, responses were classified into three ranges: low variability for standard deviation lower than 1, medium variability for standard deviation higher than or equal to 1 for the low category or lower than 2, and high variability for standard deviation higher than or equal to 2. Finally, the averages with high variability (>2) were adjusted or validated using references that have worked with similar methods [22, 53, 54]. In addition, the results obtained from expert consultations and those taken from a review of the relevant literature were subject to correlation analysis.

### 2.5 Spatiotemporal analysis of the supply of ecosystem services

The calculated potential supply of ES is based in the landscape structure, i.e., it is determined by the extension of each structural land cover and the respective individual capacity to provide particular ES. The supply of ES was obtained by multiplying the relative area of each structural land cover in 1996 or 2011by the single value in the assessment matrix of ES obtained after the expert consultation. Using those values, the supply of ES was mapped for each year. This analysis reports changes in the potential supply of each of the ES over a 15-year period. The potential supply and changes in the supply of ES were calculated separately for cultural, provisioning, and regulating services. Finally, to evaluate synergies and trade-offs between ES, all land cover changes were compared in terms of variations in the area and the corresponding effect in the ES supply.

## 3 Results

### 3.1 Structural land cover changes (1996–2011)

**Fig 3:**
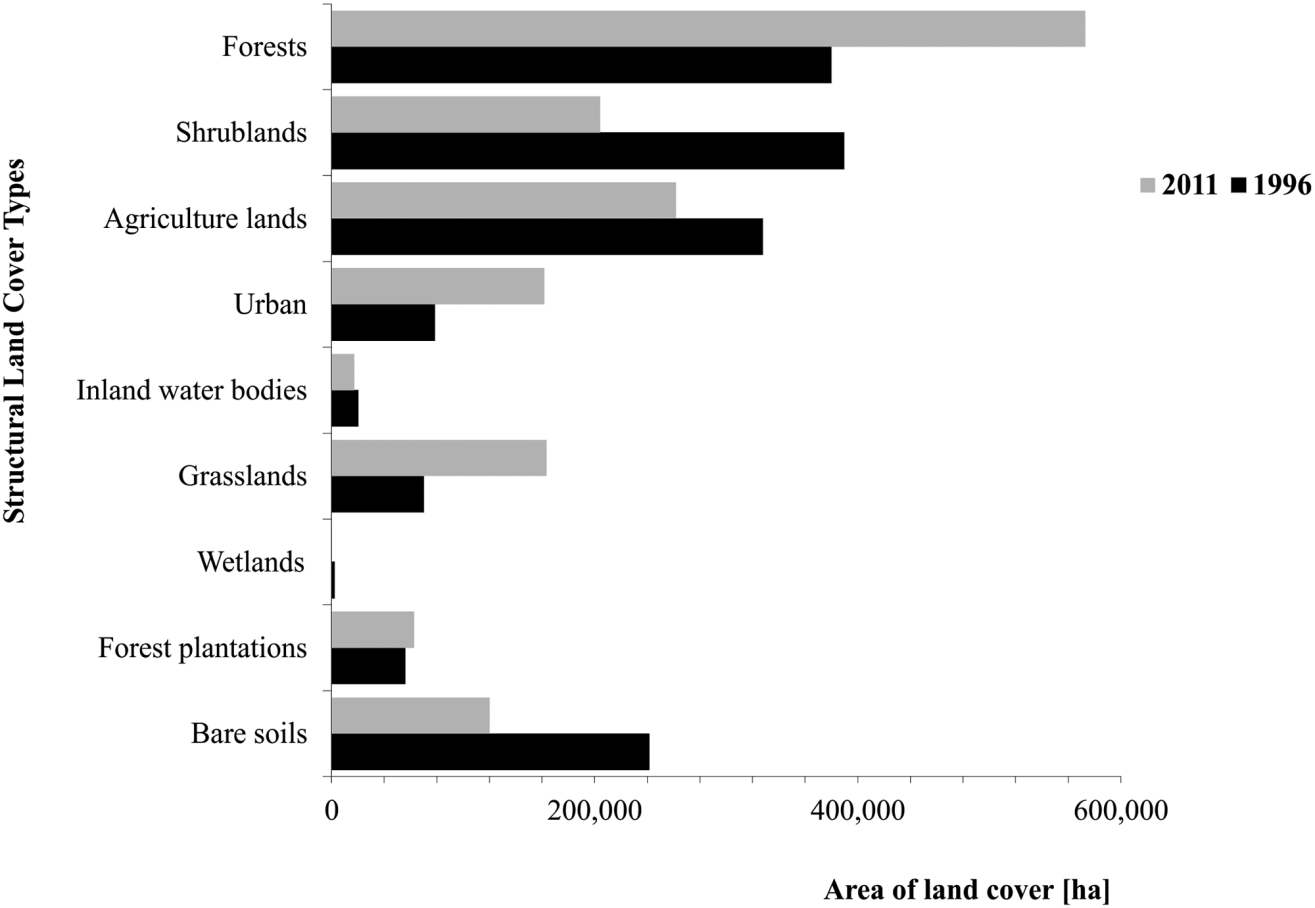
Structural land cover changes between 1996 and 2011.

The changes in structural land covers between 1996 and 2011 in terms of the absolute area and participation in the total urban region surface were significant. The following two types of changes were found. a) Increases in the surface of four structural land covers (Fig 3): 1) 193,237 hectares in forests, (+50.9%); 2) 93,484 hectares in grasslands (+130%); 3) 83,287 hectares in urban areas (+106%); and 4) 6,666 hectares in forest plantations (+12%) b) Decreases in the surface of five structural land covers (Fig 3): 1) 185,256 hectare in shrublands (-47.6%); 2) 121,142 hectares in bare soils (-50%); 3) 65,858 hectares in agricultural land (-20.1%); 4) 2,863 hectares in inland water bodies (-14%); and 5) 1,555 hectares in wetlands, representing a 75% change with respect to its surface in 1996 The most relevant changes were the increase in forests and the decrease in shrublands. Fig 3 summarizes the surfaces of each structural land cover change in the urban region for the two years.

### 3.2 Composition of changes within structural land cover types

Almost half of the urban region experienced changes in land cover, with great internal transformations in all nine structural land cover types. The biggest changes occurred in three structural land cover types. For agricultural lands, 41% of those present in 1996 changed to other land covers (mainly urban (57,745 ha)) in 2011 and, as a result, 18% of the agriculture land became urban in 2011. For shrublands, a net of 68,798 ha of shrublands in 1996 was converted to forests in 2011. For bare soils, 12,913 hectares classified as bare soils in 1996 were grasslands in 2011.

### 3.3 Expert assessment of supply of ecosystem services

**Fig 4:**
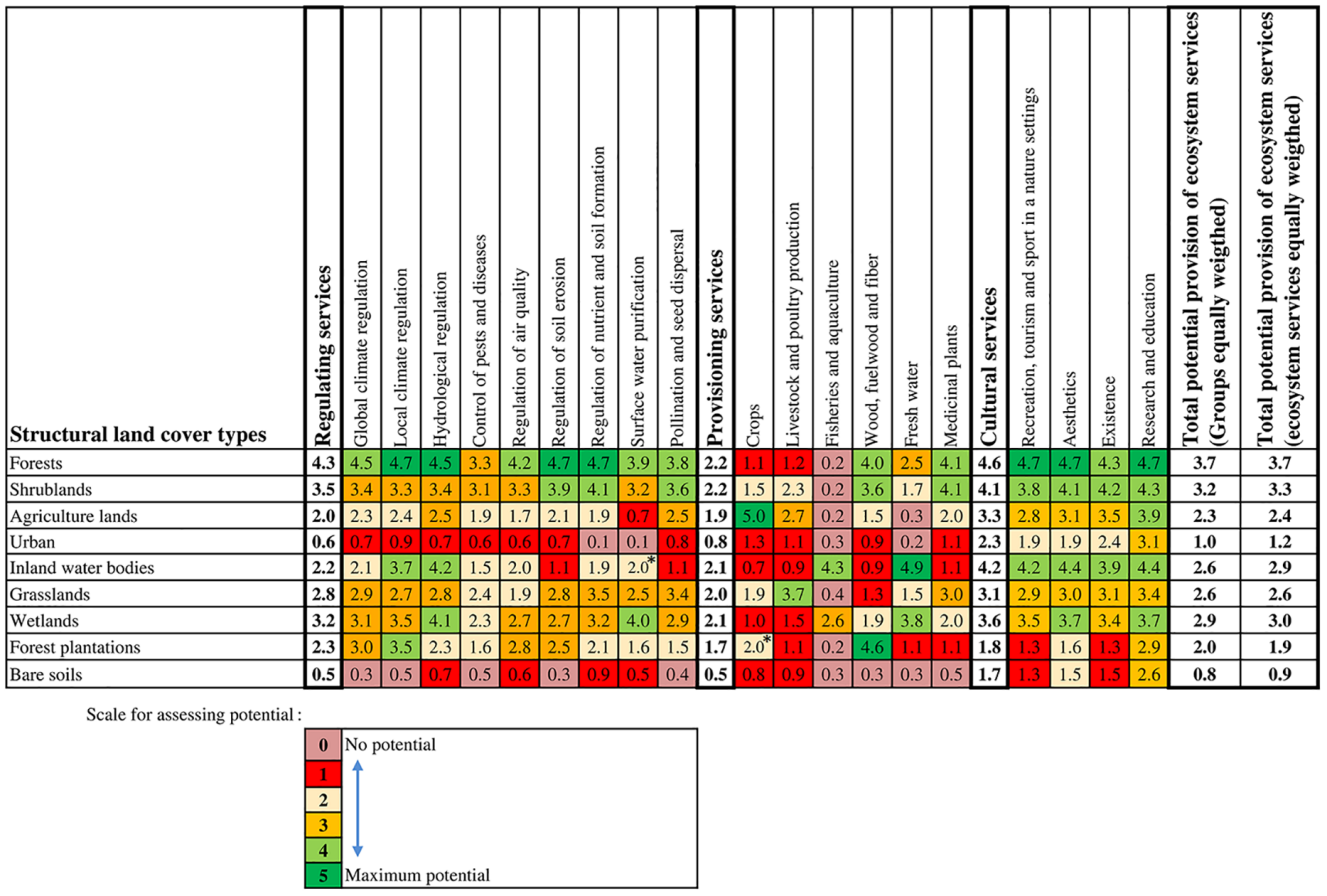
Matrix assessment of the regional ecosystem services indicating the potential of each land cover class for the provision of ecosystem services selected for the study. Score ranges considered for evaluation are an adaptation of Burkhard [53,54]. *Scores that had high variability in the assessment given by the experts.

The results obtained from the expert evaluations are shown in Fig 4. No significant variations were found between the two different weighted criteria to obtain the total potential provision of ES. Forest, covering 37% of the urban region in 2011, is the structural land cover type with the greatest potential for providing ES. Experts scored forest ES over four for regulating services (regulation of local climate, regulation of soil erosion, and regulation of nutrient and soil formation) and cultural services (recreation, tourism, and sports in a natural setting; aesthetics; and research and education). On the other hand, the highest scores for provisioning services were associated with agricultural lands for crop production and inland water bodies for the provision of fresh water (covering 17% and 1% of the urban region, respectively, in 2011). The lowest scores (0–1) for regulating services and provisioning services were primarily associated with bare soils and urban areas (7.7% and 10.3%of the urban region in 2011, respectively), which, according to expert opinion, neither regulate climate or air nor control erosion. They also do not supply timber, wood fuel or fiber, fresh water, medicinal plants, or fish.

The results from the correlation analysis, which compared the scores given by experts with scores extracted from similar studies, showed similar trends with little differences (Table 3). The exceptions are differences found in the potential provision of shrublands, which could be attributed to difficulties in comparing the existent ecosystems with those in the reviewed studies. The urban structural land cover type also showed a low correlation, probably because several studies included “discontinuous urban fabric” and, in the present study, experts only scored continuous urban fabric.

**Table 3:** Correlations between results obtained for ecosystem services valuation for each combination of ecosystem services and land cover according to the expert consultants and reviewed literature [22, 5,3, 5,5, 5,6]. Wetlands and forest plantations were not correlated because of the lack of data from the analyzed studies. Scores vary from 0 to 5, where 0 means no potential and 5 means maximum potential.

Unit of analysis	Correlation between values given by the experts and the literature reviewed (r Pearson)	Scores of ecosystem services provision given by the experts (average)	Scores of ecosystem services provision from similar studies (average)
Urban region	**0.65**	**2.32**	**2.09**
**Structural Land Cover Types**			
Forests	0.84	3.7	3.5
Shrublands	0.77	3.2	1.8
Agriculture lands	0.57	2.3	2.1
Urban	0.51	1.0	1.1
Inland water bodies	0.81	2.6	2.5
Grasslands	0.64	2.6	2.4
Bare soils	0.68	0.8	1.1
**Ecosystem Services Groups**			
Regulating services	0.66	2.3	2.1
Provisioning services	0.63	1.8	1.5
Cultural services	0.43	3.2	2.9

### 3.4 Mapping the supply of ecosystem services

**Fig 5:**
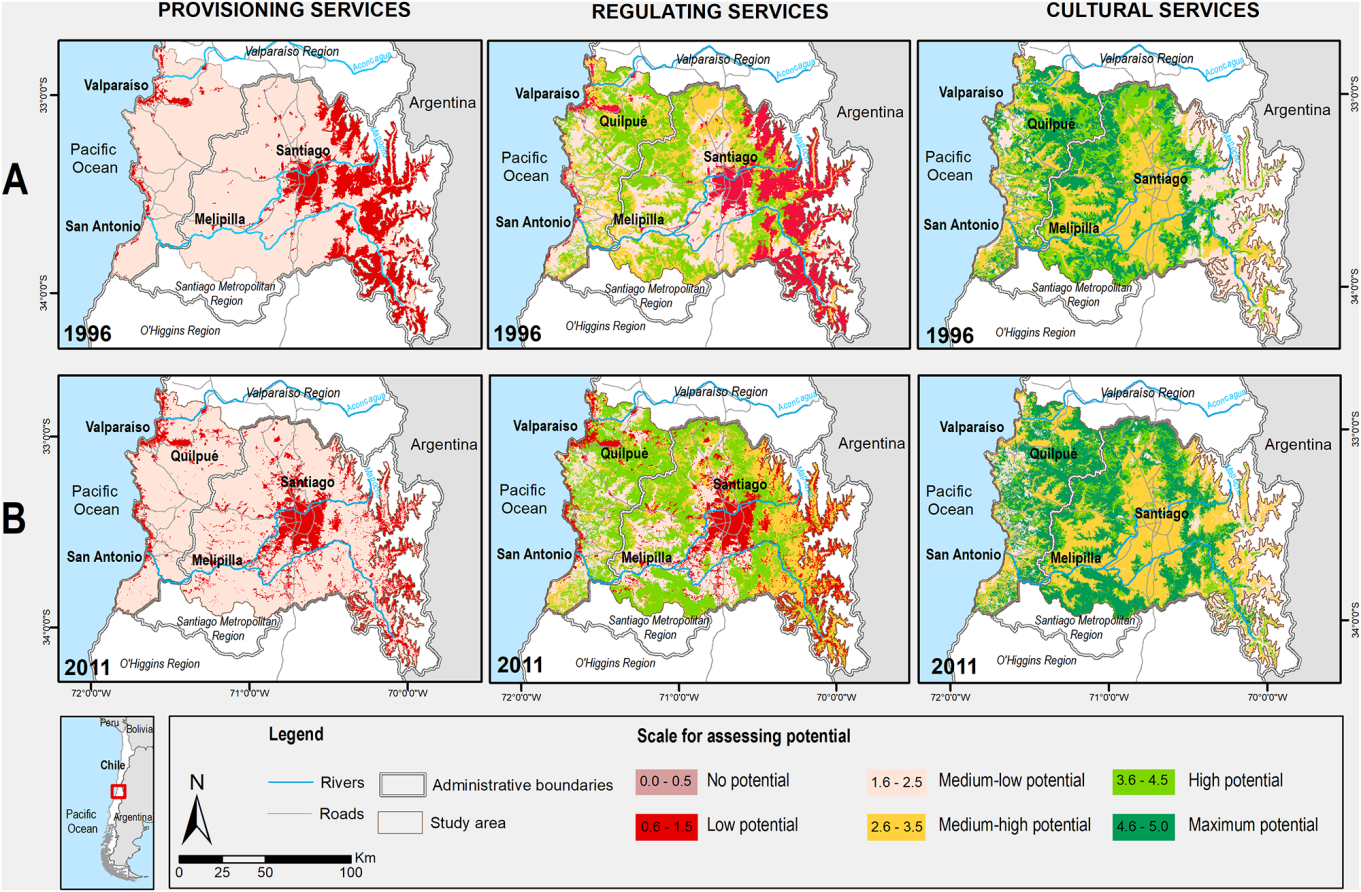
Maps of the supply of provisioning, regulating, and cultural ecosystem services in the urban region in 1996 (A) and 2011 (B).

The spatial representation of the ES supply in 1996 (Fig 5A) shows a large area with low potential for supplying services around Santiago, especially on the eastern side near the Andean foothills and, to a lesser extent, towards the west (Valparaíso), mainly in the coastal mountain range. This low supply is primarily associated with bare soils. In the central valley, areas of medium-low potential are related to dominant agricultural production activities (agriculture and forestry), whereas areas with high potential, mainly associated with forests, are located in the north of the valley. By 2011 (Fig 5B), the supply of regulating services had decreased in areas of low and medium-low potential in the Andes. Areas with medium-high potential increased, mainly owing to natural grasslands. High potential areas dominated the central valley and the coastal areas, where forests and shrublands occupied a larger territory.

Regarding provisioning services in 1996, areas with low potential were mainly concentrated on the eastern side, close to the Andes. However, by 2011, these zones were widely dispersed over the central valley near Santiago, constituting the new peri-urban areas. These areas also increased westward around Valparaiso but decreased in the Andes. The opposite effect was observed for cultural services. In 1996, the urban region was mostly dominated by medium-high potential areas, except for the eastern side, close to the Andes, where the areas of medium-low potential were located. The areas of high and maximum potential had virtually the same spatial representation in the central valley and coastal range. However, the situation changed by the year 2011; areas with maximum potential had increased and those with medium-high potential stayed the same, mainly owing to agriculture. None of the areas fell in the range of no potential for supplying cultural ES.

**Fig 6:**
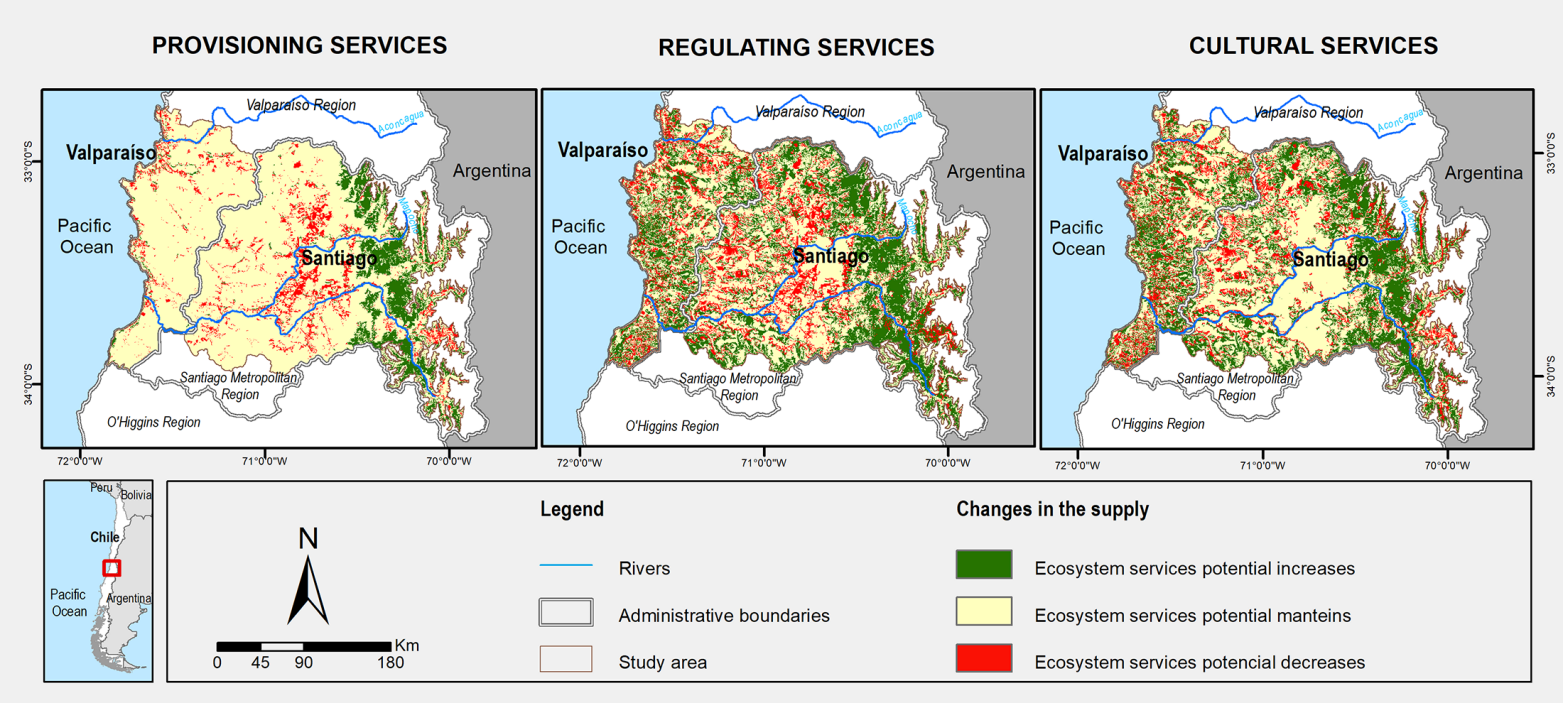
Maps of changes in the supply of ecosystem services in the urban region between 1996 and 2011.

Fig 6 shows the increases and decreases in the supply of each of the three groups of ES between 1996 and 2011. Within the provisioning services group, most of the surface (more than 80%) remains unchanged during the period. Only an approximate 10% of the urban region experienced decreases in the provision of ES, and these changes were dispersed in the central zone of Santiago and in the western area (Valparaíso), mainly towards the coast. The increase in the provision of ES was mostly apparent in Santiago, extending toward the foothills of the Andes. For regulating services, 52% of the urban region did not change, 31% increased and was dispersed throughout the area, mostly towards the Andes, whereas the remaining 17% decreased. The cultural services group increased in approximately 30% of the surface area, but decreased in 15% of the total area. For the supply of cultural services, 55% of the area remained unchanged.

### 3.5 Changes in the supply of ecosystem services for the urban region

As a result of land cover changes, the overall supply of ES increased slightly between 1996 and 2011, from 2.6 to 2.7 (medium potential) in the urban region, although ES groups changed at varying levels during the analyzed period (Table 4).

**Table 4:** Potential supply of ecosystem services in the urban region. 0 means no potential and 5 means maximum potential of supply of ecosystem services.

Ecosystem services group	Potential supply in 1996	Potential supply in 2011	Qualitative scale of supply
Regulating services	2.64	2.83	Medium-high potential
Provisioning services	1.79	1.84	Medium-low potential
Cultural services	3.44	3.57	Medium-high to high potential
Total	2.62	2.75	Medium-high potential

**Fig 7:**
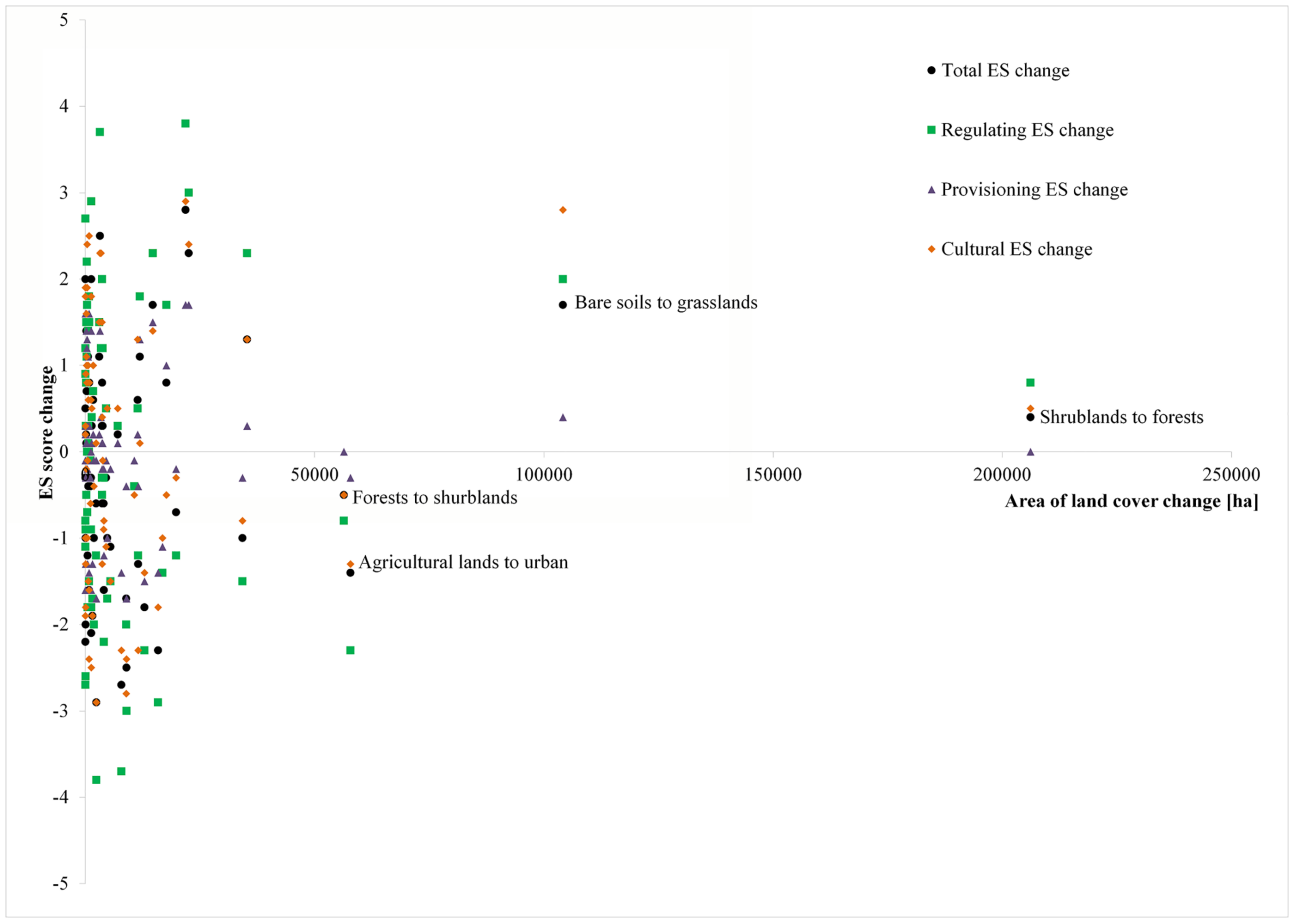
Synergies and trade-offs of ES score changes and land cover changes.

Fig 7 shows the changes in the supply of ES that the most extreme score changes were associated with regulating services. Changes in structural land covers that represent major changes in the supply of ES occurred in a reduced area of the landscape, mostly in areas smaller than or equal to 10,000 ha. Contrastingly, changes produced for larger surfaces caused slight variation in the potential supply of ES (by total or by group). The major change in terms of surface comes from shrublands to forests, representing a smaller change in the score of ES supply, as well as changes from agricultural land to urban and from forests to shrublands. The change from bare soils to grasslands affects mostly cultural and regulating ES. This implies that ES provision changed smoothly in response to the large land cover changes.

## 4 Discussion

### 4.1 Land cover changes in the urban region

The urban region showed significant transformations in terms of land cover changes compared to those reported in similar studies of Europe (e.g., [64]). However, the results are similar to other cases concerning Latin America (e.g., [3, 59]). Table 5 shows the equivalence in structural land covers in comparison to other similar studies. The nine structural land cover types include land covers present in other studies [22, 53, 55, 56]. Other land covers included in this study, such as wetlands and forest plantations, are absent from similar reviewed studies. This indicates the high capacity of this method to be adapted to local ecological configurations.

**Table 5:** Equivalence between structural land cover types used in this study and those in similar studies [22, 5,3, 5,5, 5,5].

This research	Burkhard et al., 2014	Kopperoinen et al.,2014	Koschke et al., 2012	Vihervaara et al.,2010
Forests	Broad-leaved forest Mixed forest Coniferous forest	Conservation areas Important forest habitats Important bird areas (IBA) Old forests (age C120 years)	Broad-leaved forest Mixed forest Coniferous forest	Broad-leaved forest Mixed forest Coniferous forest
Shrublands	Transitional woodland shrub Sclerophyllous vegetation		Transitional woodland-shrub	
Agriculture lands	Annual and permanent crops Permanently irrigated land Fruit trees and berries Complex cultivation patterns Vineyards Olive groves Non-irrigated arable land	Traditional agricultural biotopes	Land principally occupied by agriculture Fruit trees and berry plantations Complex cultivation patterns Non-irrigated arable land	Agricultural areas
Urban	Continuous urban fabric Discontinuous urban fabric	Sealed surfaces Discontinuous urban fabric	Continuous urban fabric Discontinuous urban fabric	Artificial surfaces
Inland water bodies	Water bodies, watercourses	Surface waters of high or good ecological status	Watercourses, water bodies	Running water Stillwater
Grasslands	Natural grassland	High nature value farmlands	Natural grasslands	Grasslands and moors
Wetlands				Marshes, bogs
Forest plantations				Forestry area
Bare soils	Beaches, dunes, and sand plains Bare rock Mineral extraction sites		Mineral extraction sites	Sand, bare rocks, etc.

### 4.2 Land cover classification methodological issues

The use of official databases is a good starting point for monitoring land cover changes. The land cover dataset used in this study provides a national coverage that has been useful for land use planning and for landscape-related sciences [39, 65]. Its limitations are low comparability between different administrative regions, which are updated in different years, and hindrance of temporal comparisons owing to slight methodological improvements [51]. Consequently, in the current research, the 66 land cover classes were standardized and merged into nine structural land cover types. This simplified approach allowed the experts consulted to take into account the scale of the analysis, breadth and depth of land cover taxonomy, differences in the supply of ES by land cover type, and dominant ecosystems in the urban region. Other studies have analyzed a higher number of land cover types and landscape elements (e.g., n = 19 for [22], n = 23 for [55], n = 44 for [54],).

### 4.3 Use of expert assessment as a tool for measuring ecosystem services

The number of experts involved in the evaluation is a crucial aspect for this kind of assessment. In this study, 15 experts were asked to assess the provision of ES. They were diverse in terms of scientific disciplines, experienced, and had expertise in the assessed landscape. Fifteen experts are sufficient for evaluation purposes, as evidenced by other studies (e.g., 13 experts in [55]; 14 experts in [58]; 14 experts in [66]; 13 experts in [67]). Previous studies have applied linear scales from 0 to 100 (e.g. [22]), 0 to 5 (e.g. [53, 54]), or -3 to 3 (e.g. [55]). In the current study, a linear scale from 0 to 5 was used to denote the relevance of the potential supply of ES, where 0 means no potential and 5 represents maximum potential. Table 6 compares the assessment features between the present study and other published work.

**Table 6:** Experts consulted, land covers, and ecosystem services used in this and other similar studies [22, 5,3, 5,5, 5,6].

Author	Spatial scale	Number of experts	Number of land covers	Number of ecosystem services	Land covers		Ecosystem services	
					Shared land covers	Land covers included by some authors, but not in this research	Shared ecosystem services	Ecosystem services included by some authors, but not in this research
Burkhard et al., 2014	Regional	NA	44	31	21	23	22	9
Kopperoinen et al., 2014	Regional	13	23	21	9	14	12	9
Koschke et al., 2012	Regional	12	19	13	13	6	10	3
Vihervaara et al., 2010	Local	20	13	23	11	2	12	11
This research	Regional	15	9	19	-	-	-	-

The standard deviation (SD) of the expert responses shows no large discrepancies between the experts. Only 1.7% of the scores had high variability (SD ≥ 2). As expected, opinions were not unanimous; 62% of scores had median variability (1.1 < SD < 2), whereas 36.3% had low variability (SD ≤ 1). By group, approximately 35% of the results for provisioning and regulating services had low variability, whereas 42% of the results for cultural services had low variability. For the results with high variability, three groups of ES had a percentage below 3%, whereas a large percentage for regulating and provisioning services (over 60%) had medium variability. The experts also did not fully agree on cultural services, as more than half of the results (55.6%) were distributed in the medium range of variability.

The answers given by the experts varied greatly for three interactions between land covers and ES: (1) the role of inland water bodies in the purification of surface water, (2) the contribution of monoculture forest plantations to crop production, and (3) the role of bare soils for research and education. We reduced our experts’ scores from 3 (medium-high potential) to 2 (medium-low potential) for interactions (1) and (2) and kept the original scores for interaction (3) based on our review of the literature. The experts consulted had the chance to score each land cover in a range from 0 to 5. Although they considered that urban land cover can provide regulating and provisioning services, they scored it very low (<1.0). In some cases, the experts gave scores higher than 0, probably acknowledging activities such as urban agriculture in the case of crops in urban areas (only 6/15 experts scored this ES as 0).

The group of provisioning services received quite low scoring owing to the low values the experts gave to most of the provisioning services. The experts provided these scores, considering the hypothetical maximum performance of each land cover to supply each ES, based on their own expertise. The low response variability for interactions between land covers and ES revealed the absence of strong disagreements, despite experts had varying degrees of knowledge on the possible links between ES and land cover classes [55].

Expert assessment of regulating services for bare soils might have been underestimated; these services can be higher than those offered by rocky outcrops (i.e., participation in global climate or local weather). Additionally, beaches and dunes could be good resources for tourism and could participate in the regulation of nutrients and water, neither of which were taken into account. Therefore, the assessment of these services might be higher than the scores given in our study. To provide a more detailed assessment, it would be necessary to link these specific land cover classes with onsite measurements of regulating and cultural services.

Evaluations of ES though methods based on expert assessment make possible to evaluate several ES simultaneously, providing a holistic view of landscape changes. The greatest challenge for this method is contrasting the results with selected biophysical indicators, as illustrated in the work of Burkhard [54], and their treatise on energy supply and demand. Strong correlations between indicator-based and expert-based data were found in other studies [22].

### 4.4 Changes in the long-term provision of ecosystem services

The slight increase in the provision of ES in the Santiago-Valparaiso urban region is remarkable, especially in the context of the ongoing, larger landscape transformations. Other studies have found little variation for a German region (Saxony; [60]) and significant declines in two Latin American regions, El Salvador, [3] where changes in ES are mainly provided by tropical forests and coastal wetlands, and the Colombian Amazon, which has shown different trends for each group of ES, provisioning services being the most affected [32]. In Santiago-Valparaiso, the majority of land covers provide little ES, which should be considered a warning and duly accounted for in land use planning. Regarding regulation services, the spread of forest plantations and the appearance of forest patches could partially explain the increase. Processes linked to disperse urbanization can strengthen cultural services. When looking for well-being and comfort, whether environmental, social, or economic, people move closer to environmentally valuable ecosystems and subsequently generate new sources of cultural services, particularly aesthetics, existence, and recreation [68]. For cultural services, the main changes were associated with transformations from bush to forests. Bare soils were transformed into grasslands in the Andes. There were no significant changes in productive land use, as reported in official datasets; however, in the early 1990s, during a period of approximately seven years, scarce snowfall generated a higher elevation of zero isotherm and decreased the availability of water resources in the following dry seasons [69, 70]. As a consequence, the vegetation land cover decreased, i.e., grasslands dried up. With the return of snow in 1997 and continuing snowfall in the following years [69, 70], vegetation regrowth and bare soils became grasslands again. This climatic variability mostly affected areas with high variability in vegetation coverage, such as those located in the piedmont, thereby explaining some relevant ES provisioning changes with regard to the role of bare soils in the assessment.

**Fig 8:**
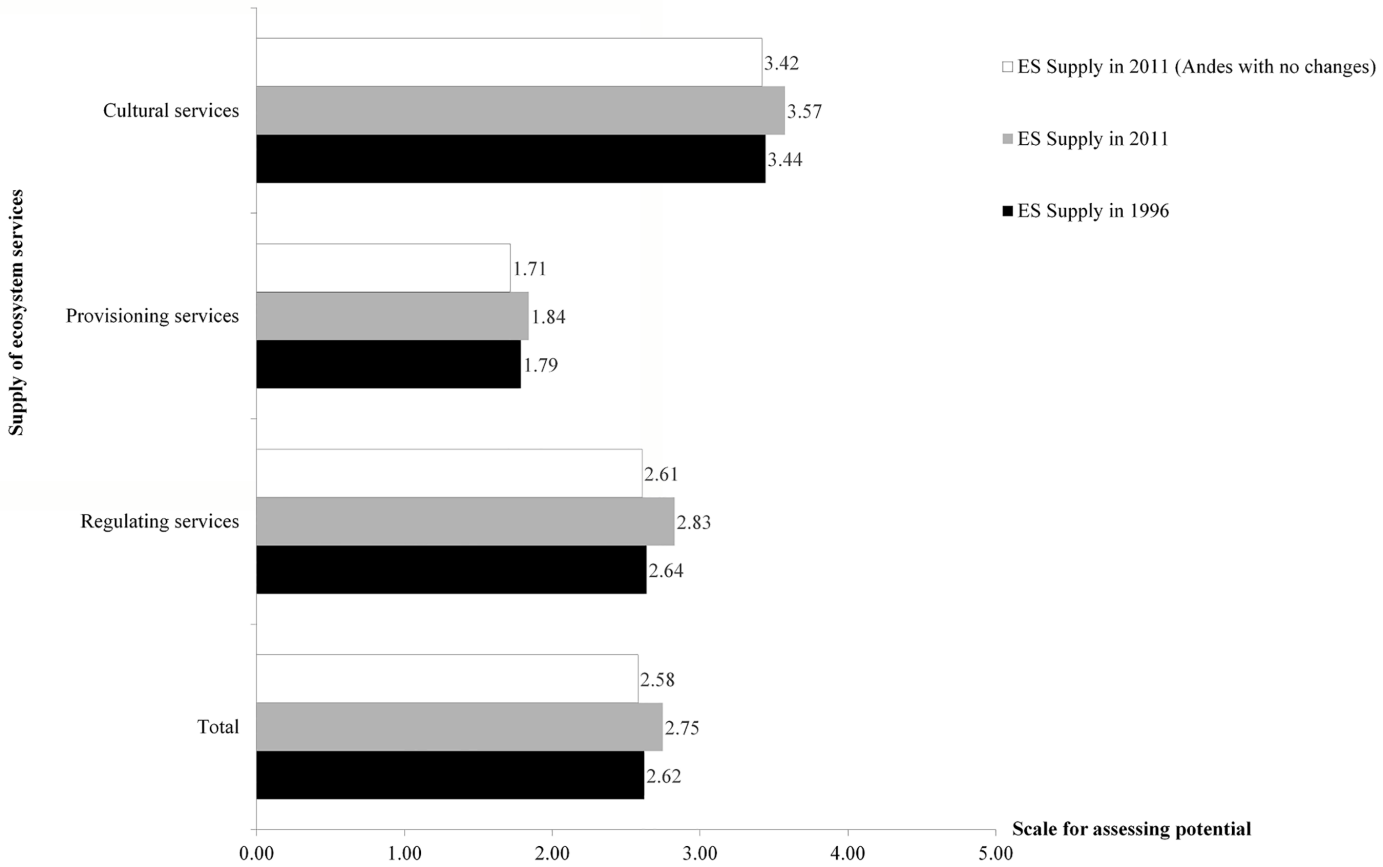
Supply of ES in the urban region if the Andes had not changed (1996–2011).

Because the results obtained include changes in the Andes, we calculated what would have been the supply of ES if the Andes had not changed. The Andean area was delimited using the height elevation where the Andean piedmont ends and the mountainous area (1,200 m.a.s.l.) begins. The results show that changes in the Andes affected the potential supply of ES in the entire urban region (Fig 8). The initially reported increase in the ES supply for all ES and its groups now showed a decrease.

### 4.5 Role of land planning regulations in the supply of ecosystem services in the urban region

The significant changes observed in the landscapes of the Santiago-Valparaíso urban region are, in part, the result of the complex and confusing legal framework and the regulations implemented ad-hoc with differential effects on the land use (Table B in S1 Appendix). Regional Metropolitan Plans lay down rules to control some landscape changes, such as establishing rules determining where projects and infrastructure can be developed and creating protected environmental areas (i.e., natural ecosystems) and protected agricultural areas, most of which restrict urban development. Whereas municipal land use plans only regulate the urban areas, other specific regulations have greater impact on the urban region and restrict compliance with, and the functionality of, land use planning. For instance, regulations such as the law for the division of rural plots (D.F.L. 3516 of 1980) have fostered urbanization outside the urban limits established in municipal land use plans, allowing residential development in the countryside, which typically includes large permanent or temporary residences built on plots of up to 0.5 hectares. This regulation profoundly changed the purpose of rural areas from productive, as established in land use planning, to residential, laying the groundwork for suburbanization [17]. The intricate framework of land use planning has had a strong impact on the landscape of the Santiago-Valparaíso urban region. It has led to the loss of agricultural areas, despite national efforts to strengthen the farming industry [17, 71], and a high dispersion of peri-urban housing developments. Recent peri-urbanization plays a fundamental role in the reduction of agricultural land [17, 71]. Land previously used for agriculture is now used for residential purposes (e.g., gated communities), because of land regulations that allow urbanization [17].

On the other hand, regarding the increase in ES, it might be due to increases in biomass as a result of ecological succession in protected areas and specific programs to bolster the production of agricultural products. For example, changes observed in the study, such as the replacement of shrublands by grasslands, could be associated with soil improvements resulting from a focus on animal production and grazing. However, some shrublands could be negatively affected by grazing activities and transformed into grasslands. Moreover, the strong promotion of agriculture through multiple financial and regulatory instruments has led to agricultural development on slopes, especially of avocado trees and vineyards for wine production, in the Central Valley [72]. In addition, the increase in forests could be related to methodological improvements made to the cadasters [51].

### 4.6 Implementing ecosystem services in land planning for urban regions

Different methodologies (e.g., field measurements, consulting experts, and working with stakeholders; [29, 22, 45, 58, 73, 74, 75]) have been used to quantify, assess, and map ES for land use planning. These scientific contributions have led to the inclusion of ES in land use planning and management [19, 45, 76]. However, relevant methodological gaps remain for putting the ES framework into practice in land use planning [77]. Furthermore, researchers must use robust databases to analyze the provision of ES, a feature currently lacking in many countries [27, 29, 22, 57, 58]. Several strategies exist for incorporating the ES framework into land use planning and management [76]. Two feasible options for urban regions in Latin America are: 1) to evaluate the effects of land regulations using development scenarios linked to specific changes in ES provision (e.g., [60, 78]) and 2) to set standards or quantitative targets for an appropriate proportion of the regional landscape that should have high or maximum relevant potential for supplying ES. Land cover types, as presented in this study, can greatly help in setting up the aforementioned environmental goals.

In Chile, none of the existing land use planning regulations have incorporated the concept of ES, nor the necessary requirements for ensuring their long-term supply. The present study found that the supply of ES at the regional scale has slightly increased, despite the major changes occurring in the peri-urban and rural areas of the Santiago-Valparaiso urban region. This is a positive result. However, because such increase is not a direct result of land planning, it cannot be ensured that the supply of ES will be maintained in the future. There is a lack of awareness of the relevance of ES changes, as emphasis is mostly given to the economic aspects, at the expense of the environment. Hence, there is a strong need for better coordination of regional and municipal land use planning to ensure the protection of areas with high ES provision, by regulating the immense pressures posed by economic development. Land planning must ensure the minimum amount of ES necessary to achieve landscape sustainability [19, 30, 79]. This can be accomplished by studying certain land cover types and monitoring trends in economic, natural, and social changes to protect them. Forests, shrublands, and water bodies are the areas making greater contributions to the supply of ES. Public concern should be focused on developing appropriate land use planning tools to enhance, consolidate, and protect these land cover types.

## 5 Conclusions

In this study, land cover changes and the supply of ES have been linked, providing an assessment of landscape transformations in a highly dynamic urban region. This research presented an adapted method based on the use of official land cover datasets and expert consultation to assess the potential supply of ES in an urban region. Land cover types were grouped into structural land cover classes to facilitate the temporal comparison of combined datasets from different administrative regions with slight methodological inconsistencies. Applying this method allows a spatiotemporal analysis of landscape transformations and their respective effects on the provision of ES.

The results describe specific losses and gains of ES supply, without finding relevant changes at a larger scale. Land cover changes and their effects on the supply of ES are caused by a myriad of diverse and intricate landscape transformations governed by a wide set of regulations, policies, land management practices, economic drivers, etc. Thus, to manage the supply of ES land regulations and policies, we should consider the provision of ES provided by specific land cover types. The results can directly inform land policy for better management of the ES supply in the studied region. Additionally, decision-making can be greatly improved by including spatiotemporal monitoring for ex post evaluations of land regulations and policies.

The proposed method can support the design of land use regulations by using the ES framework. It can be applied in other urban regions of Latin America and the world facing changes in ES owing to landscape transformations.

## Supporting information

S1 Table XLS file containing the main calculations of the supply of ecosystem services. (XLSX)

S1 Appendix XLS file containing the homologation of the classes, policies and regulations with effect on landscapes. (XLSX)
